# Targeting *Leishmania* Fe-SOD and Glucose Metabolism with Tripodal and Pyridinacyclophane Polyamines as a Chemotherapeutic Strategy

**DOI:** 10.3390/metabo16050322

**Published:** 2026-05-12

**Authors:** Álvaro Martín-Montes, Estefanía Delgado-Pinar, Irene Bonastre, M. Paz Clares, Begoña Verdejo, Álvaro Martínez-Camarena, Rafael Ballesteros-Garrido, Rubén Martín-Escolano, Mª José Rosales-Lombardo, Enrique García-España, Clotilde Marín

**Affiliations:** 1 Departamento de Parasitología, Facultad de Ciencias, Universidad de Granada, 18071 Granada, Spain; amartim@ugr.es (Á.M.-M.); mjrosale@ugr.es (M.J.R.-L.); 2 Facultad de Salud y Deporte, Universidad Alfonso X el Sabio-Mare Nostrum, Camino de la Térmica, 90, 29004 Málaga, Spain; 3 Departamento de Química Inorgánica, Instituto de Ciencia Molecular, Universidad de Valencia, C/Catedrático José Beltrán 2, 46980 Paterna (Valencia), Spain; estefania.delgado@uv.es (E.D.-P.); irene.bonastre@uv.es (I.B.); begona.verdejo@uv.es (B.V.); enrique.garcia-es@uv.es (E.G.-E.); 4 Departament de Química Orgànica, Universitat de València, Av. Vicent Andrés Estellés, 22, 46100 Burjassot (València), Spain; m.paz.clares@uv.es (M.P.C.); rafael.ballesteros-garrido@uv.es (R.B.-G.); 5 Departament de Química Física, Universitat de València, Av. Vicent Andrés Estellés, 19, 46100 Burjassot (Valencia), Spain; alvaro.martinez@uv.es; 6 Unidad de Infección Viral e Inmunidad, Centro Nacional de Microbiología (CNM), Instituto de Salud Carlos III (ISCIII), 28220 Majadahonda, Madrid, Spain; r.martin@isciii.es; 7 Centro de Investigación Biomédica en Red en Enfermedades Infecciosas (CIBERINFEC), Instituto de Salud Carlos III (ISCIII), 28029 Madrid, Spain

**Keywords:** *Leishmania*, glucose metabolism, polyamine, superoxide dismutase

## Abstract

**Highlights:**

**What are the main findings?**

- Polyamines as possible leishmaniasis chemotherapy.
- Targetin Leishmania Fe-SOD and glucose metabolism.

**What are the implications of the main findings?**

- Compounds are easy to synthesize and store, with high solubility and acid resistance.
- Promising properties enable oral administration.

**Abstract:**

**Background/Objectives**: Many parasitic diseases remain without an effective treatment and cause many deaths worldwide. Leishmaniasis is a complex disease that belongs to the category of Neglected Tropical Diseases, as its treatment relies on outdated drugs that also lead to resistance and negative side-effects. To address this problem, two new chemical families have been tested in vitro against three of the most common parasites from the genus *Leishmania*. **Methods**: One family is formed by the polyamine tris(2-aminoethyl)amine functionalised either in one or its three primary amines with different aryl group, and the other is a group of azamacrocyclic cyclophanes containing either one or two aromatic spacers. **Results**: From the first family, only one compound showed activity against *Leishmania donovani*, and from the second family, three compounds were selective, two of them for *Leishmania braziliensis* and a different one against *L. donovani*, another parasite of the studied genus. **Conclusions**: The anti-*Leishmania* activity seems to be related to the compounds’ ability to inhibit the iron superoxide dismutase activity and to alter the parasite metabolism by inhibiting glucose intake in *L. braziliensis* or by accelerating it in *L. donovani* and by attacking the parasite defences against ROS, both effects triggering a mitochondrial membrane depolarization that enhances damage, leading to cell death.

## 1. Introduction

Leishmaniasis, a complex disease caused by protozoan parasites of the genus *Leishmania*, represents a major global health problem [1]. It is not a simple disease due to the variety of symptoms it presents, depending on the species of the genus that infects the host. There are three main clinical traits: Cutaneous Leishmaniasis (CL), which can cause single or disseminated ulcers or lesions; Mucocutaneous Leishmaniasis (ML), which is characterized by the destruction of facial connective tissue that causes deformations; and Visceral Leishmaniasis (VL), the most threatening form of the disease due to irregular fever and hepatosplenomegaly that can lead to organ malfunction and death [2]. As mentioned above, all these forms are caused by parasites of the genus *Leishmania*, which are unicellular flagellated parasites that have two forms during their life cycle: promastigote, the infectious phase, which is fusiform, has a flagellum, and reaches a size of between 15 and 20 µm and amastigote, a round and smaller form (2 to 5 µm) that is responsible for the division of the parasite within the host macrophage cells [3].

In recent years, the need for surveillance programs has increased since the largest outbreak of the disease in Europe took place in Madrid, central Spain in 2009, with 800 cases reported [4]. After some epidemiological studies performed after the outbreak, it was found that the parasite *L. infantum* is geographically prevalent in the area due to the abundance of reservoirs like the Iberian hare (*Lepus granatensis*) and cats (*Felis catus*) [5], which raises concerns due to the finding of the presence of *Leishmania* in other domestic animals that were initially unsuspected.

Treatments for Leishmaniasis are based on outdated drugs that possess a variety of negative side effects. The most widespread treatment is based on pentavalent antimonials, such as sodium stibogluconate and meglumine antimoniate [6]. Those drugs, although effective, present a long and difficult administration regime, as they must be administered by trained personnel in health centers, a commodity that is not as ubiquitous as it should be in developing countries, which means that the patient must commute long distances regularly to be treated. On top of that, these drugs are toxic to the liver and kidneys, and can also affect the heart. For these reasons, in this article, two families of new drugs have been studied as candidates for Leishmaniasis treatments [7].

New drugs should possess some features if they are to be considered better alternatives to current drugs. An affordable price could be achieved by starting from affordable reagents using scalable synthesis; thermostability would be an interesting feature, as it would ease drug storage and distribution; lack of side effects is the most pursued feat, along with an easy administration method (for instance, an oral administration regime would be ideal for the patient to take at home).

**Figure 1:**
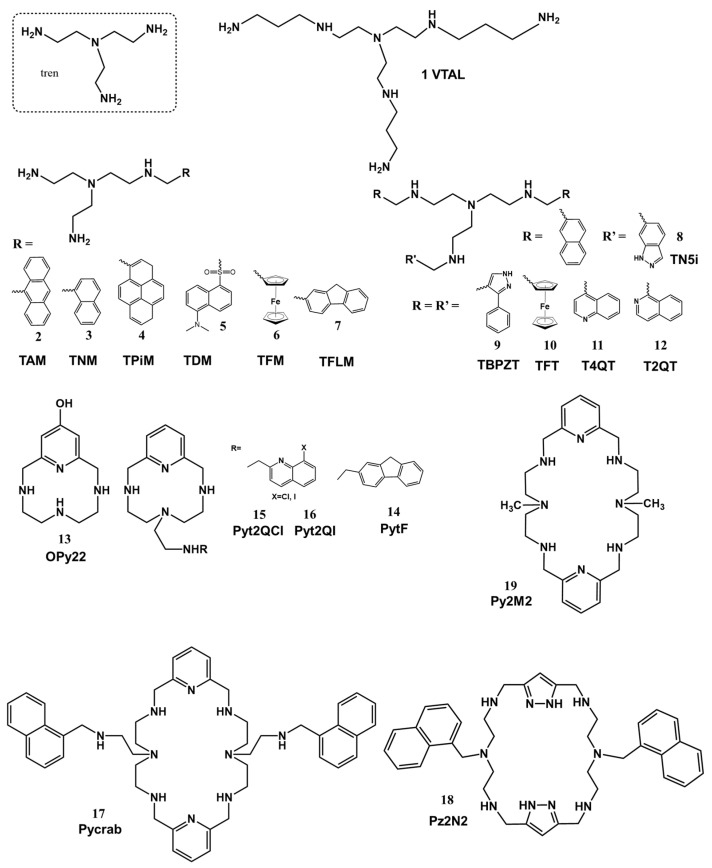
Structures of the compounds studied in this work.

Here, we report on a series of compounds belonging to two families, one formed by the polyamine tris(2-aminoethyl)amine (tren) functionalised either in one or its three primary amines with different aryl groups and the other one consisting of a group of azamacrocyclic cyclophanes containing one or two aromatic spacers (Figure 1), which have shown interesting in vitro results against three representative species from the genus *Leishmania*. Given the essential role of polyamines in parasite growth and survival and their particular relevance in trypanosomatid metabolism, these systems also represent attractive targets for the development of novel antileishmanial strategies [8].

## 2. Materials and Methods

### 2.1. Chemistry

#### 2.1.1. Synthesis and Characterization of the Compounds

General synthetic procedures: The tris(2-aminoethyl)amine (tren) derivatives (compounds 2–12, TAM (**2**), TNM (**3**), TPIM (**4**), TDM (**5**), TFM (**6**), TFLM (**7**), TN5I (**8**), TBPZT (**9**), TFT (**10**), T4QT (**11**), T2QT (**12**)) were obtained in high yields by straightforward synthetic pathways previously described [9]. The experimental procedure consisted of a two-step route: (i) reaction in dry ethanol or THF in the case of 10 (TFT) of the corresponding carboxaldehyde with the polyamine tris(2-aminoethyl)amine (tren) in an adequate molar ratio (aldehyde–polyamine 1:4 for the monofunctionalized compounds 2–7 (TAM (**2**), TNM (**3**), TPIM (**4**), TDM (**5**), TFM (**6**), TFLM (**7**)), and 3:1 for the trifunctionalized ones (TBPZT (**9**), TFT (**10**), T4QT (**11**), T2QT (**12**)) and (ii) reduction with sodium borohydride. Finally, the compounds were isolated as hydrochloride or hydrobromide salts for their conservation and further handling.

Compound **1** (VTAL) was prepared by a multistep procedure described by Albelda et al. [10]. Tosylated polyamine tren was reacted with three moles of bromopropylphthalimide to introduce a 3-aminopropyl extension in each branch. Detosylation to obtain 1 (VTAL) as its hexahydrobromide salt was performed using a mixture of HBr/HAc in phenol.

On the other hand, compounds of the cyclophane family were obtained following reported synthetic procedures. To obtain OPy22 (**13**), a slightly modified procedure from that described in [11] was employed. Then, 4-benzyloxy-2,6-bis(chloromethyl)pyridine was reacted in CH_3_CN with the pertosylated amine. The protecting groups were removed with a HBr/HAc mixture to obtain the macrocycle as a hydrobromide salt.

**Scheme 1:**
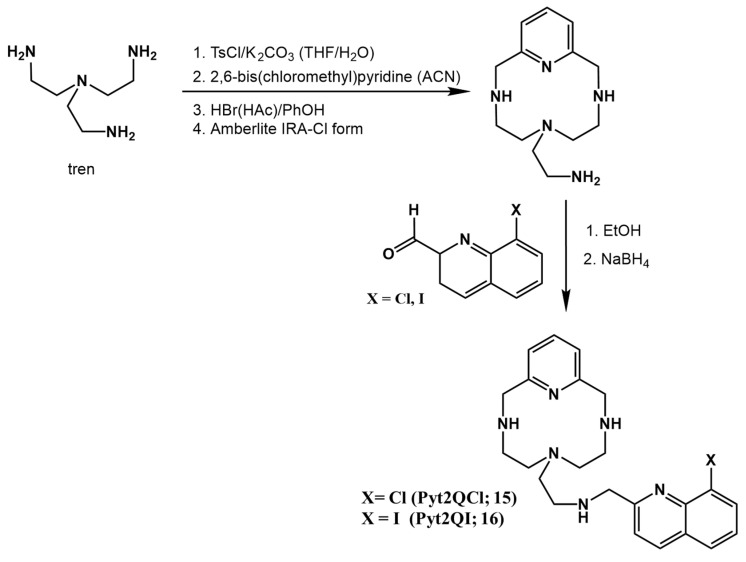
Synthesis of Pyt2QCl (**15**) and Pyt2QI (**16**).

The novel compounds Pyt2QCl (**15**) and Pyt2QI (**16**) were obtained in a similar way by reacting pertosylated tren(2-aminoethyl)amine with 2,6-bis(chloromethyl)pyridine in EtOH followed by detosylation with a HBr/HAc mixture (see Scheme 1). The resulting hydrobromide salts were transformed into the free amines using an Amberlite IRA 402 ion exchange resin [12], and they were then reacted with the corresponding carboxaldehyde followed by reduction with sodium borohydride. Details of the synthesis of the new compounds are provided below (Scheme 1).

PyCrab (**17**) was obtained by reacting TNM (**3**) with 2,6-pyridine dicarbaldehyde in EtOH in equimolar amounts followed by reduction with sodium borohydride [13]. To obtain Pz2N2 (**18**), 1H-pyrazole-3,5-dicarboxaldehyde was added to a solution of 3-(naphthalene-2-ylmethyl)pentane-1,5-diamine in MeOH in a stoichiometric amount. After workup, the compound was isolated as a hydrochloride salt [14]. Py2M2 (**19**) was prepared by a similar route but in this case by reaction of 2,6-pyridine-dicarboxaldehyde with 2,2′-diamino-N-methyldiethylamine in ethanol. The compound was isolated after reduction with sodium borohydride as its hexahydrobromide salt.

All compounds were characterized by NMR spectroscopy and produced satisfactory elemental analysis.

Synthesis of Pyt2QCl•4HCl (**15**): Briefly, 6-(2-Aminoethyl)-3,6,9-triaza-1(2,6)-pyridinacyclodecaphane (0.58 g, 2.32 mmol) and 8-chloroquinoline-2-carboxaldehyde [15] (0.45 g, 2.35 mmol) are dissolved in 100 mL of dry ethanol and stirred at room temperature for 2 h. Then, sodium borohydride (0.9 g, 23 mmol) is added, and the bulk is stirred for 2 h more. The solution is vacuum-evaporated and extracted with CHCl_3_/H_2_O (3 × 100 mL). The organic phase is taken to dryness and dissolved in dry ethanol. The hydrochloride salt of the product is precipitated by adding a concentrated hydrochloric acid solution. Yield: 68% ^1^H NMR (400 MHz, D_2_O) δ 8.50 (d, *J* = 8.5 Hz, 1H), 8.05–7.95 (m, 3H), 7.69–7.60 (m, 2H), 7.48 (d, *J* = 7.8 Hz, 2H), 4.75 (s, 2H), 4.66 (s, 4H), 3.61–3.50 (m, 2H), 3.30 (d, *J* = 8.4 Hz, 6H), 2.99 (s, 4H). ^13^C NMR (75 MHz, D_2_O) δ 152.0, 148.9, 139.8, 139.0, 131.7, 130.6, 129.1, 127.6, 127.5, 122.2, 120.8, 51.1, 50.5, 49.5, 45.9, 43.0. Elemental analysis: calculated for C_23_H_26_N_6_Cl·4HCl·2H_2_O: C: 45,75%; N:13.92%; H: 5.67%. Found: C: 45.84%; N:14.02%; H: 5.26%
Synthesis of Pyt2QI·4HCl (**16**): The same procedure applies for the synthesis of Pyt2QCl, but 8-iodoquinoline-2-carboxaldehyde is used instead of 8-chloroquinoline-2-carboxaldehyde. Yield: 74% ^1^H NMR (500 MHz, D_2_O) δ 8.51 (d, *J* = 7.4 Hz, 1H), 8.40 (d, *J* = 8.4 Hz, 1H), 8.05 (d, *J* = 8.2 Hz, 1H), 7.99 (t, *J* = 7.9 Hz, 1H), 7.57 (d, *J* = 8.5 Hz, 1H), 7.46 (dd, *J* = 17.0, 8.0 Hz, 3H), 4.77 (s, 2H), 4.66 (s, 4H), 3.62 (t, *J* = 7.9 Hz, 2H), 3.39–3.27 (m, 6H), 3.02 (t, *J* = 5.3 Hz, 4H). ^13^C NMR (126 MHz, D_2_O) δ 148.9, 140.9, 139.2, 129.2, 128.5, 122.2, 120.4, 51.3, 50.64, 49.4, 46.0, 42.9. Elemental analysis: calculated for C_23_H_26_N_6_I·4HCl·4H_2_O·0.4(C_4_H_8_O_2_): C: 38.54%; N:10.96%; H: 5.42%. Found: C: 38.54%; N:11.02%; H: 5.08%

#### 2.1.2. Methodology of the Computational Studies

The computational study of the ligands started with the building of their structures employing the xleap software included in AMBER16 (version 17.06) [16] (Assisted Model Building with Energy Refinement) software (AmberTools 17.6). The protonation degree of the ligands at physiological pH and the distribution of the protonated amines along the receptors were deduced by analysis of the speciation studies: H_4_Pycrab^4+^ and H_2_Pyt2QCl^2+^. The representative structure of the Fe-SOD enzyme was taken from the Protein Data Bank (PDB ID: 2GPC) [17,18]. Although the model used (2GPC) corresponds to the FeSOD of *Trypanosoma cruzi*, it was selected because it is a close structural homologue of the FeSOD from *Leishmania major*, as evidenced by comparisons between the chosen structure (2GPC) and the corresponding PDB entry (4F2N). To build up the L:Fe-SOD complexes, the ligands were approximated to the active center of the enzyme using LEaP (AmberTools 17.6).

Once the systems were built, they were energetically minimized and, after an equilibration stage at 300 K, a total of 10 ns (10 cycles of 106 steps each) molecular dynamics (MD) was performed. During the modelling, a distance restraint was applied between the heavy atoms of the active site residues and the heavy atoms of the compounds, using the ⟨r-6⟩-1/6 average of all the interaction distances to atoms of the groups. All the studies were done using AMBER16 [16] software. The organic compounds were modelled using the gaff [19] force field while the ff14SB [20] one was used for the Fe-SOD. Finally, a series of 10 minimum energy conformers were selected and then energetically optimized again. The MD simulation trajectory was analyzed using the cpptraj module [21] within AmberTools17.6, while PyMOL 1.7.7.2 [22] was employed for visual inspection and to create the molecular graphics.

### 2.2. In Vitro Biological Evaluation

The following experiments were conducted in order to study drugs’ leishmanicidal activity against three *Leishmania* species. A screening parasite strategy was coordinated with cytotoxicity assays against J774.2 macrophages. The resulting IC_50_ and Selectivity Index (SI) addressed the selection of active compounds, and then a first approach to elucidate their action mechanism was carried out, assessing whether drug activity could be explained by Fe-SOD inhibition, metabolic alterations or mitochondrial membrane depolarizations, or a combination of those.

#### 2.2.1. Parasite Strain and Culture

Parasites were cultured in medium trypanosome liquid at 27 °C in axenic cultures and standard conditions. This culture medium was synthetized in our laboratory according to [23].

The selected strains were *L. infantum* (MCAN/ES/2001/UCM-10), *L. braziliensis* (MHOM/BR/1975/M2904) and *L. donovani* (MHOM/PE/84/LC26) [24]. Parasites were stored at −80 °C before being cultured.

#### 2.2.2. In Vitro Activity Assays

Before use in the assays, the compounds were dissolved in dimethyl sulfoxide (DMSO, Panreac, Barcelona, Spain) and added to macrophage cell cultures or to extracellular and intracellular forms of the parasite to achieve the required final concentrations. The concentration of DMSO in the assays never exceeded 0.01% (*v*/*v*).

#### 2.2.3. Cytotoxicity Assays

Macrophages from the J774.2 cell line were used as model for host cells. [European collection of cell cultures (ECACC) number 91051511]. They were cultured in minimal essential medium (MEM) plus glutamine (2 mM) and supplemented with 20% inactive FCS and then stored at 37 °C in a humidified atmosphere consisting of 95% air and 5% CO_2_.

Compound cytotoxicity was determined by flow cytometry according to a method described in [24]. Viable cell percentage was calculated with respect to the control culture. The IC_50_ values were calculated using linear regression analysis from the K_c_ values of tested concentrations (100, 50, 25 and 12.5 μM).

#### 2.2.4. Compound Activity on Extracellular Parasite Forms

As a quick and easy first approach for compound antiparasitic activity, an assay against extracellular forms of *Leishmania* (called promastigotes) was performed. Compounds were added to the culture medium in 24-well microplates (Nunc) in order to reach final concentrations of 12.5, 25.0, 50.0 and 100.0 µM. After 72 h, parasites were counted using a Neubauer haemocytometric chamber. Compound activity is expressed as IC_50_ concentration, as this is the concentration needed to achieve a 50% inhibition in the cell population.

#### 2.2.5. Compound Activity on Intracellular Parasite Forms

Macrophages were first cultured in 24-well microplates (Nunc) with round coverslips at the bottom, where they will attach. The initial cell concentration was 10^4^ macrophages/well. Then, they were infected with promastigotes at a 10:1 ratio. At the same time, compounds were added to the medium to reach the same concentrations as in the promastigote experiment. Promastigotes underwent conversion to amastigotes one day after infection. After two days, coverslips were collected and fixed to a microscope slide. For their examination on optic microscope, Giemsa staining was needed [24].

#### 2.2.6. Determination of Compound Effectiveness

Cytotoxicity and extracellular and intracellular results were collected in the previously described experiments. To determine if a compound should be evaluated further as a drug candidate, one should use the Nwaka criteria: an IC_50_ close to 10 µM and a Selectivity Index higher than 20 times the one obtained with the reference drug. Selectivity Indexes (SIs) were calculated by dividing the macrophage IC_50_ by the parasite IC_50_. If a compound fulfils Nwaka’s criteria, it will be considered active, and its effects on infectivity, SOD inhibition, metabolic alterations and mitochondrial membrane alterations will be evaluated [25].

#### 2.2.7. Effects on Infectivity

With this experiment, the objective is to determine if a compound affects the parasite’s ability to infect cells and to divide within them.

The methodology was the same as in the amastigote assay, although this time the test dosage was IC_25_. Tested drugs and parasites were added at the same time. After the initial 12 h, wells were washed with fresh culture medium to remove any promastigotes that did not infect macrophages. Coverslips were collected after 2, 4, 6, 8 and 10 days after infection, as it is assumed that infection reaches its peak after 10 days.

Two parameters were measured: infection rate (percentage of infected cells) and amastigote number per infected cell.

### 2.3. Action Mechanism Approach

The following experiments were performed in order to determine the reason for compounds’ anti-leishmanial activity.

#### 2.3.1. SOD Enzymatic Inhibition

Oxidative burst is the cell prime defence method when confronting an infection. Trypanosomatid parasites have evolved to counteract this mechanism by developing an enzyme known as superoxide dismutase (SOD), the first of many enzymes responsible for scavenging the toxic reactive oxygen species (ROS) that will be generated after the oxidative burst. Trypanosomatid parasites depend on SOD to survive inside the infected cell and then divide. One way a compound could be effective is by inhibiting this enzymatic activity and rendering the parasite defenceless against ROS [24].

In addition to its dependence on this enzyme, what makes SOD an ideal molecular target is the fact that in *Leishmania*, it is bound to an iron atom (Fe-SOD) and in vertebrates to a copper and a zinc atom (Cu/Zn-SOD) or to a manganese atom (Mn-SOD), which makes them different enzymes with the same function.

Enzyme quantity was determined by the Bradford method and enzyme activity by the Beyer and Fridovich method [26,27].

Cu/Zn-SOD was purchased from Sigma-Aldrich^®^ (St. Louis, MO, USA), and Fe-SOD was obtained in our laboratory by subjecting the parasites to stress in a culture medium without calf serum supplements, following the method described in [28].

#### 2.3.2. Metabolic Alterations Test

Trypanosomatid parasites do not metabolize glucose to CO_2_ and H_2_O as mammals do. Instead, they excrete part of the carbonated skeleton to the medium. As this carbonated skeleton can take the form of different metabolites, measuring these metabolite quantities and comparing them to the control conditions can indicate if there is a metabolic alteration taking place in a particular stage of the metabolic route. To discover if a compound triggered a particular metabolic effect, 10^4^ promastigotes were cultured in conical-bottom tubes using compounds’ IC_25_ concentrations as tests dosages for 72 h. Later, they were centrifuged at 400× *g* for 10 min, and supernatants were collected and analyzed via ^1^H NMR [29].

#### 2.3.3. Mitochondrial Membrane Depolarization Evaluation

The mitochondrial membrane is a key structure for the parasite, which also makes it a target for potential drugs. Sometimes results for metabolic alterations and mitochondrial membrane depolarization are correlated. Any drop in mitochondrial membrane potential will result in a malfunction and even in cell death.

The methodology of this experiment was the same as that of the ^1^H NMR experiment described above, using IC_25_ as a test dosage, but this time the pellet was collected and washed three times with PBS. After washing, the pellets were re-suspended with rhodamine (Rho 123) 10 µg/mL in PBS and left to react for 20 min and then analyzed by flow cytometry [30].

## 3. Results and Discussion

### 3.1. Antiparasitic Activity

Surprisingly, none of the compounds showed biological activity against *L. infantum* (Table 1). However, some compounds belonging to the cyclophanes family showed antileishmanial activity: Opy22 (**13**) and Pyt2QCl (**15**) were active against *L. braziliensis* (Table 2) and PyCrab (**17**) only against *L. donovani* (Table 3). In the case of the polyamine tren family compounds, TFM (**6**) stands out for its promising activity and specificity against the *L. donovani* parasite (Table 3).

Thus, four compounds—Opy22 (**13**), Pyt2QCI (**15**), TFM (**6**), and PyCrab (**17**)—displayed potent activity against *L. braziliensis* or *L. donovani*, with amastigote IC_50_ values close to 10 µM or SI higher than 20, a threshold established by Nwaka’s criteria [25]. Compounds that met both criteria were selected for further mechanistic studies.

### 3.2. Infectivity Assays

**Figure 2:**
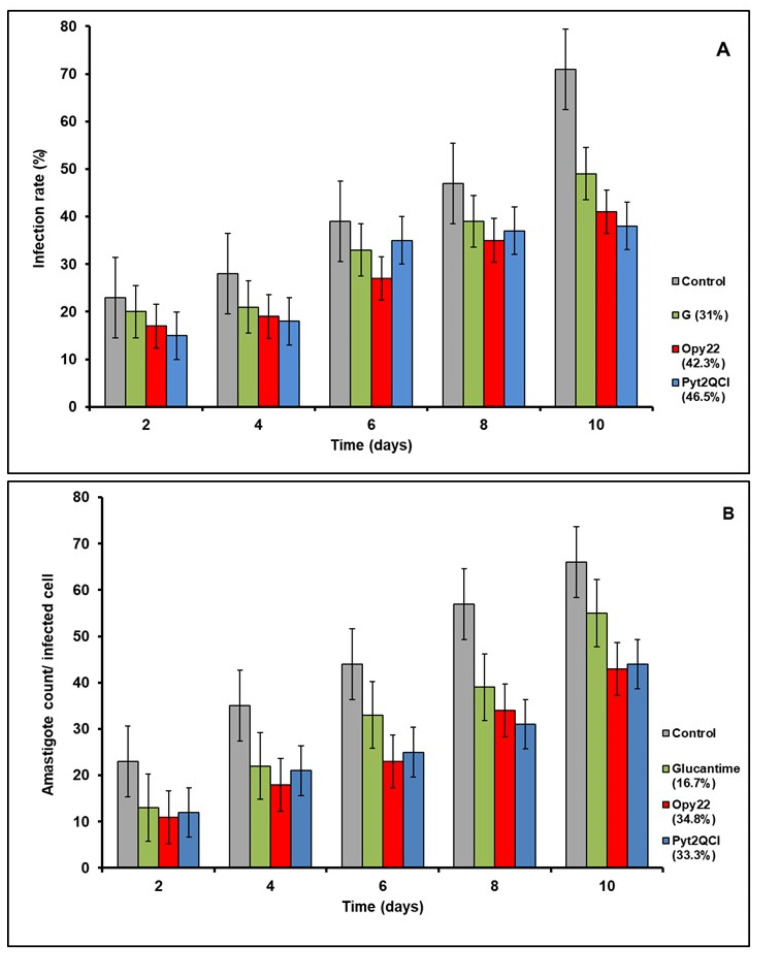
(**A**) Infection rate and (**B**) amastigote count per infected cell in *L. braziliensis* when treated with compound IC_25_ concentrations. Compounds **Opy22** (**13**) and **Pyt2QCl** (**15**) belong to the cyclophanes family. **G** stands for the reference drug Glucantime. Percentages indicate reduction in the measured parameter with respect to the control. Results are the average of three independent experiments.

**Figure 3:**
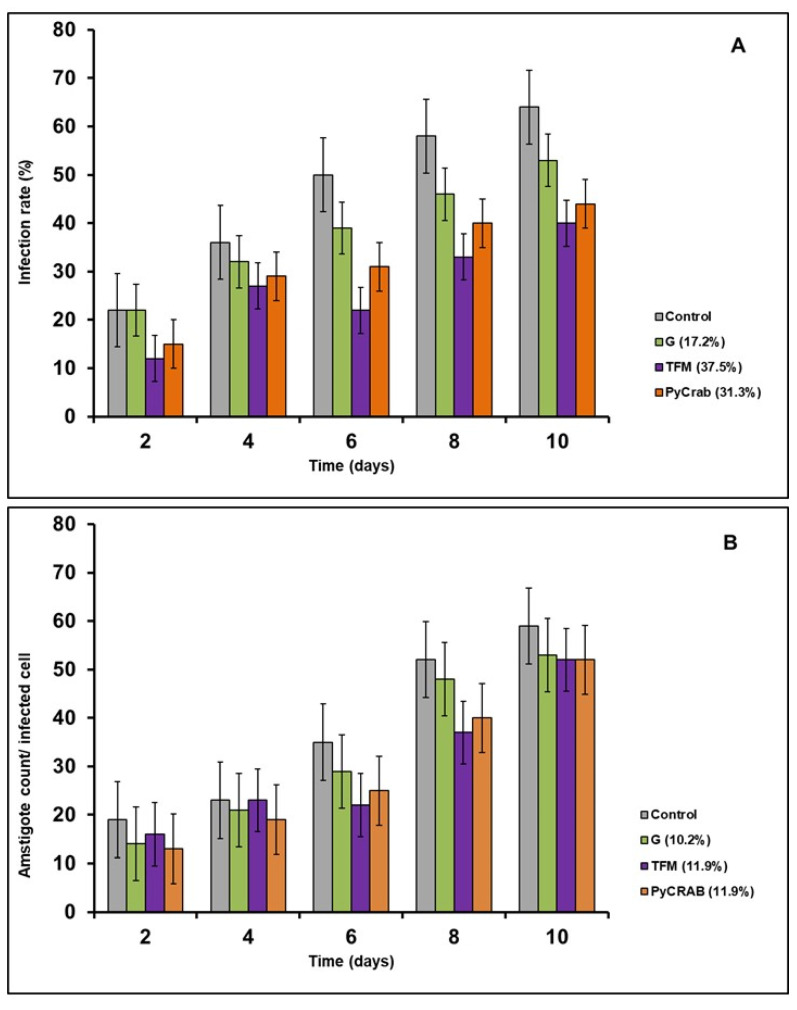
(**A**) Infection rate and (**B**) amastigote count per infected cell (**B**) in *L. donovani* when treated with compound IC_25_ concentrations. Compound **TFM** (**6**) belongs to the tren family. Compound **PyCrab** (**17**) belongs to the cyclophanes family. **G** stands for the reference drug Glucantime. Percentages indicate reduction in the measured parameter with respect to the control. Results are the average of three independent experiments.

In order to assess the effect of the compounds on parasitic infective ability, two parameters were measured: infection rate and amastigote number per infected cell. In all the experiments, the selected compounds reduced infection rate and amastigote quantity on treated cells. The achieved results surpassed the effect of Glucantime (Figure 2), which on average reduced both parameters by an average of approximately 20% except in the *L. braziliensis* infection rate experiment (Figure 2), while the drugs (the compounds tested) achieved higher results with the same IC_25_ dose. The results clearly show that the selected compounds have a greater impact on the infection rate than on the parasite’s ability to divide within the host cell. A reduction of over 30% with respect to the control in the infection rate was achieved in all cases, while the amastigote count was not reduced as much (Figure 3). Only OPy22 (**13**) and Pyt2QCl (**15**) achieved a similar reduction in the amastigote count in *L. braziliensis*, but the reduction in the infection rate was greater than this.

The conclusion is that these compounds are more effective in reducing parasite infectivity than division ability. Despite this difference, a reduction in infected cells in the population is still a promising result.

### 3.3. Effects on Fe-SOD Enzymatic Activity

**Figure 4:**
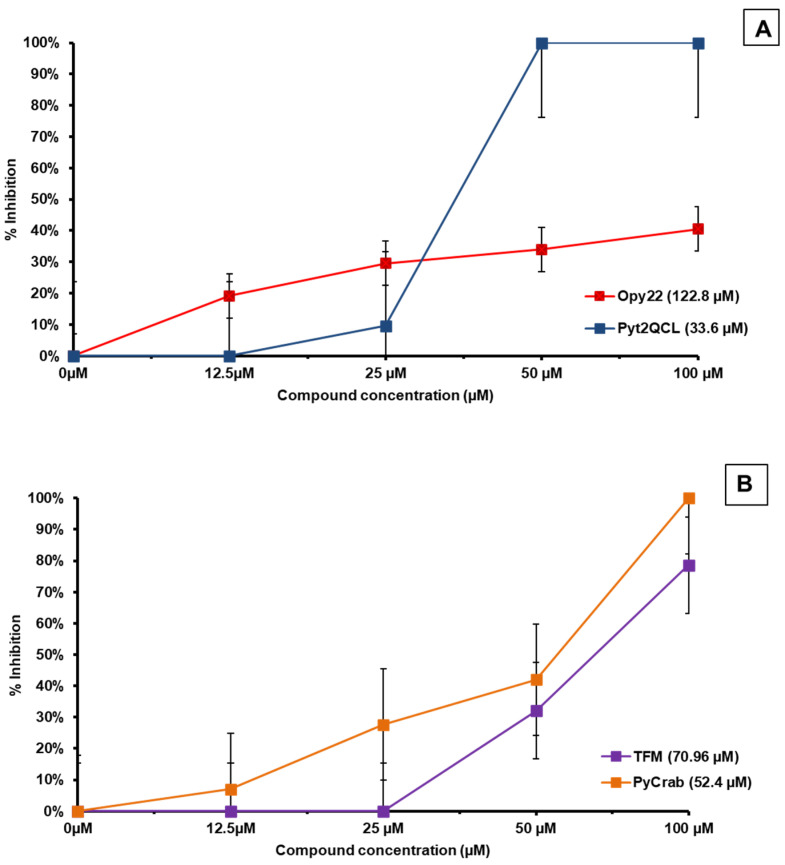
Inhibition on parasitic Fe-SOD enzymatic activity on (**A**) *L. braziliensis* and (**B**) *L. donovani*. Numbers in parenthesis show the concentration at which 50% enzyme inhibition was achieved (IC_50_). Results are the average from three independent experiments.

The selected compounds proved effective when inhibiting parasitic Fe-SOD activity, which could explain their anti-leishmanial activity. Two compounds totally inhibited the enzymatic activity at the highest concentration. Another noteworthy fact is that, in general terms, inhibition values are slight when treated with low concentrations and begin to be more noticeable from 25 µM in most cases, or even from 50 µM, as is the case with TFM (**6**) and, even more so, with Pyt2QCl (**15**), which inhibited all enzymatic activity at that concentration (Figure 4).

Compounds also proved to be selective, as they showed no inhibition when tested against human Cu/Zn-SOD, with no inhibitory effect even at high concentrations. This could explain their low cytotoxicity against macrophages.

### 3.4. Computational Studies

**Figure 5:**
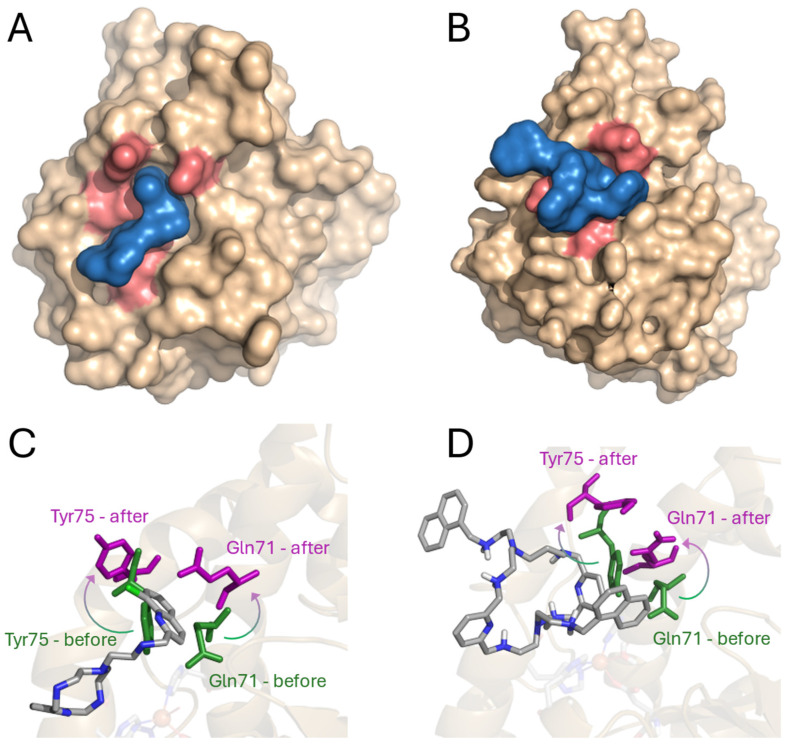
Surface representation of Fe-SOD enzyme during its interaction with (**A**) Pyt2QCl (**15**) and (**B**) PyCrab (**17**) and detail of the interaction with (**C**) Pyt2QCl (**15**) and (**D**) PyCrab (**17**). The structure of the protein is colored in pale brown; the active center surface is depicted in pink; and the studied compounds are shown in blue. In (**C**,**D**), the configuration of Tyr75 and Gln71 before the interacion with the polyamines has been depicted in green, while the configuration after the interaction has been colored in pink.

**Figure 6:**
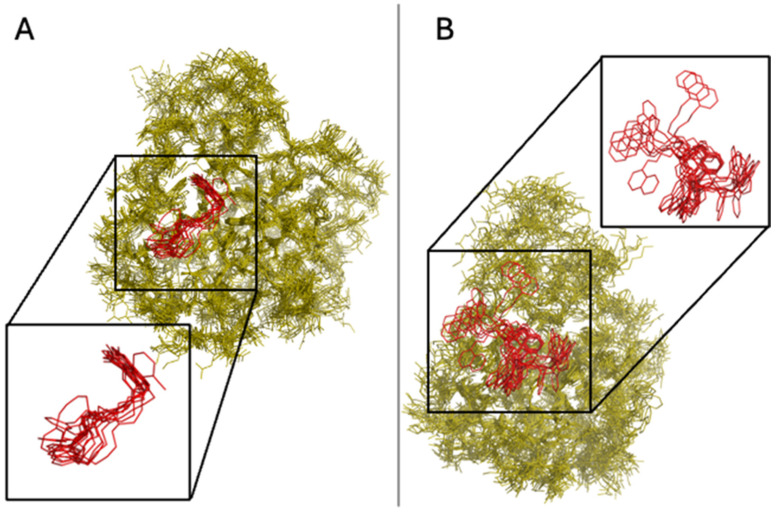
Superimposition of the ten minimum energy structures when (**A**) Pyt2QCl (**15**) and (**B**) PyCrab (**17**) interact with the model of Fe-SOD. The structure of the protein is represented in yellow, while the ligand is colored in red in both cases.

**Figure 7:**
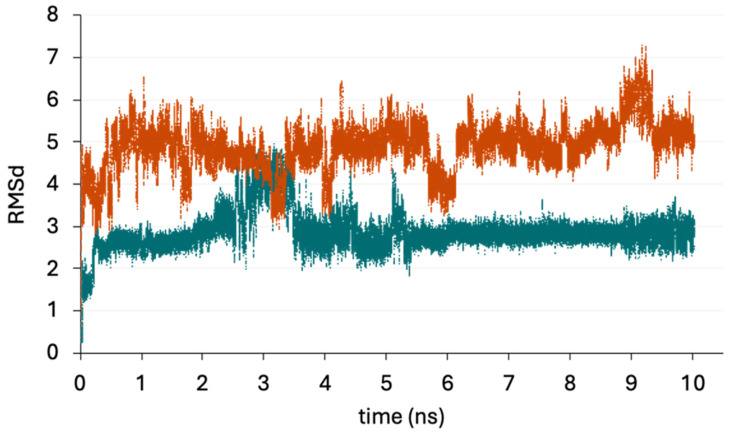
RMSd of Pyt2QCl (**15**) (in blue) and PyCrab (**17**) (in orange) as a function of the time of calculation (ns).

**Figure 8:**
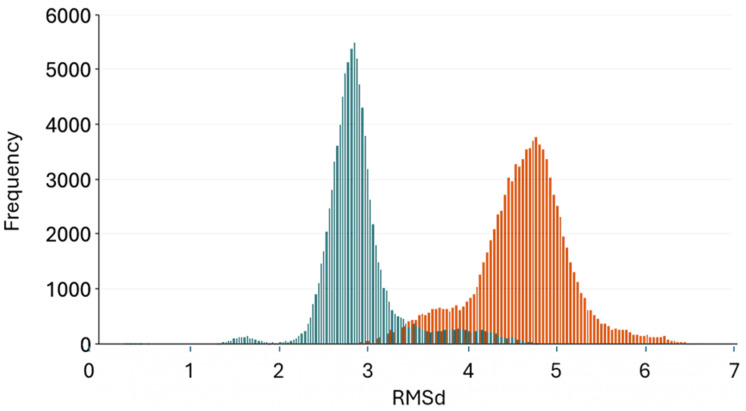
Histogram of RMSd frequencies of Pyt2QCl (**15**) (in blue) and PyCrab (**17**) (in orange) as a function of the time of calculation (ns).

To understand the effect of the studied compounds on the enzymatic activity of Fe-SOD, the interaction between the active center of the enzyme and two representative ligands was modelled using molecular mechanics–molecular dynamics (MM-MD) methods. For this, compounds PyCrab (**17**) and Pyt2QCl (**15**) were chosen and placed in the proximity of the active center of Fe-SOD. After a minimization and an equilibration stage at 300 K, a 10 ns molecular dynamics study was performed. The results of the modelling are depicted in Figure 5, Figure 6, Figure 7 and Figure 8.

Before analyzing the theoretical results, close attention should be paid to the Fe-SOD active center. Three components make up the Fe-SOD active site: (a) an iron atom, which drives the activity of the enzyme; (b) a water molecule coordinated to the metallic center; and (c) nine amino acid residues, each of which has a distinct function in the activity of the enzyme. The amino acids in the active site can be classified into three main groups based on their role in the catalytic mechanism: (a) amino acids coordinated with the metal ion (His27, His75, Asp160, and His164); (b) amino acids that form the “gateway” to the active site funnel (His31 and Tyr35); and (c) amino acids that participate in the hydrogen bond network (Tyr35, Gln71, Asn74, and Trp124). Although the model used (2GPC) corresponds to the FeSOD of *Trypanosoma cruzi*, it was selected because it is a close structural homologue of the FeSOD from *Leishmania major*, as evidenced by comparisons between the chosen structure (2GPC) and the corresponding PDB entry (4F2N). In addition, our group has extensive experience working with this protein. We are currently adapting our studies to the 4F2N mode.

The investigated polyamines present a good affinity to the Fe-SOD active center. As shown in Figure 5, both PyCrab (**17**) and Pyt2QCl (**15**) fit perfectly into the catalytic pocket, interacting with both the residues coordinated to the metal and with those of the gateway of the active center funnel, especially with Tyr75 and Gln71. The interactions of both compounds cause a clear distortion of the structure of the native enzyme. However, significant dissimilarities between the interaction of Pyt2QCl (**15**) and PyCrab (**17**) arise upon closer analysis.

The main difference between the ligand interactions with the active center of the Fe-SOD lies in the manner in which the interaction takes place and the extent of ligand binding. Pyt2QCl (**15**) interacts in its entirety with the enzyme: the macrocycle interacts with the innermost part of the active center through ionic and hydrogen bonds, whereas the aromatic quinoline ring interacts through pi-stacking interactions with the amino acids Tyr75 and Gln71 of the gateway, displacing them. In contrast, only the PyCrab (**17**) macrocycle interacts with the amino acids of the active center, while the naphthalenes are free, not establishing any clear bond with the enzyme.

This can be clearly seen in Figure 6, in which the 10 minimum energy conformers of the trajectory are overlapped. While the Pyt2QCl (**15**) structures fit perfectly over each other, especially as far as the quinoline ring is concerned, those of PyCrab (**17**) show a much larger dispersion. In fact, in the latter case, the naphthalene ring presents a very different arrangement in each conformer, which shows the limited importance of the latter during the interaction with the enzyme. This conclusion is reinforced if we analyze the root-mean-square deviation (RMSd) of the polyamine compound for the trajectory, depicted in Figure 7 and Figure 8. The RMSd value for Pyt2QCl (**15**) is 2.9 ± 0.4; in the case of PyCrab (**17**), this value increases to 4.9 ± 0.6, which emphasizes the higher mobility of the latter and therefore its lower affinity for the Fe-SOD active center.

The different interaction modes of Pyt2QCl (**15**) and PyCrab (**17**) with Fe-SOD allow us to draw some conclusions regarding the key elements to take into account in order to optimize such an interaction (i.e., the size and the linearity of the polyamine). The Fe-SOD presents a relatively small access tunnel to the active center. Therefore, only compounds with rather small dimensions are able to fully interact with it. This is the case for Pyt2QCl (**15**), which has a 12-membered macrocyclic ring and is thus able to interact with the residues closest to the metal ion of the Fe-SOD without blocking the interaction of the quinoline with the gateway of the enzyme. In contrast, PyCrab (**17**) has a much larger 24-membered ring. By interacting via hydrogen and ionic bonds with the amino acids of the Fe-SOD active center, it completely occupies the Fe-SOD gateway, preventing the naphthalenes of its own structure from interacting with the enzyme in a manner equivalent to that of the quinoline in Pyt2QCl (**15**).

### 3.5. Effects on Parasite Metabolism

**Figure 9:**
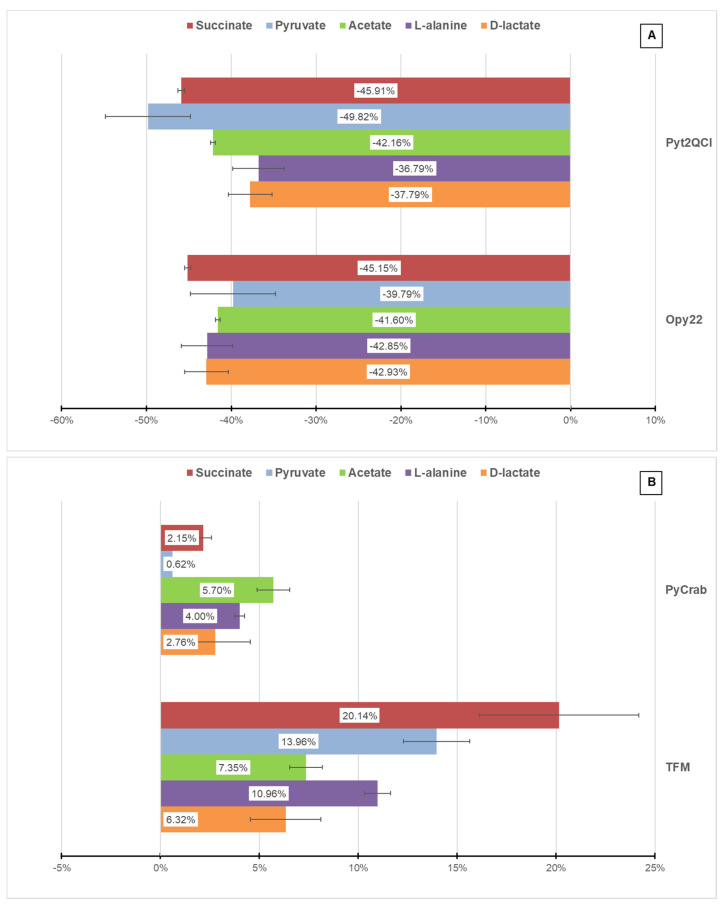
Effects on metabolite production and excretion (increase or reduction) with respect to the control in *L. braziliensis* (**A**) and *L. donovani* (**B**). Metabolites were detected in culture medium supernatants. Results are the average of three separated determinations.

Compound effectiveness can be linked to the impossibility of extracting energy from the medium by the parasites, depriving a necessary enzyme of their metabolic route, or the acceleration of the metabolism, which may lead to nutrient exhaustion of the medium. The ^1^H NMR spectra show that these compounds have an effect on parasite glucose metabolism. In general terms, both compounds analyzed in *L. braziliensis* (Figure 9A) showed an inhibitory effect due to lower metabolic excretion, which was approximately 40% lower than the control. This could be explained by a possible interaction of the compounds with glucose intake or perhaps by glucose being prevented from being processed properly, leading to starvation of the parasites. On the other hand, results obtained in *L. donovani* (Figure 9B) increase the metabolite production and subsequent excretion of metabolic end-products resulting from glucose metabolism, which could suggest metabolic acceleration, which could lead to nutrient exhaustion. These results suggest that the compounds may interfere with membrane glucose channels, either opening or closing them.

### 3.6. Effects on Mitochondrial Membrane Potential

**Figure 10:**
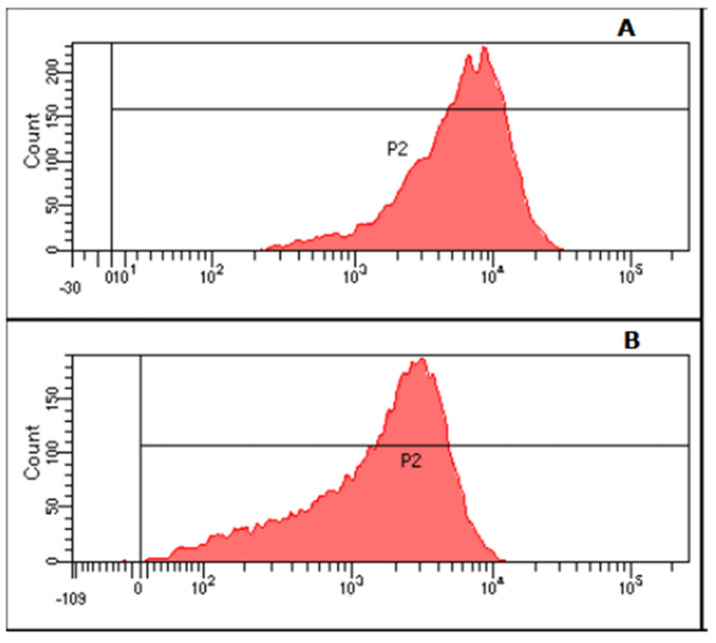
Flow cytometry charts obtained for *L. braziliensis* control (**A**) and Pyt2QCl (**15**) treatment (**B**) using IC_25_ as test dosage.

Mitochondrial membrane potential was measured to determine whether compound activity is associated with membrane depolarizations. A control experiment (Figure 10A) was carried out for comparison with the tested compounds. Mitochondrial membrane potential was unaffected by almost every tested compound; for this reason, with the exception of *L. braziliensis* membrane potential when treated with Pyt2QCl (**15**) (Figure 10B), where a slight depolarization was observed. This depolarization may reduce parasite’s ability to absorb glucose from the medium, thereby compromising its survival, as shown in previously described experiments.

These results show the promising activity of Opy22 (**13**) and Pyt2QCI (**15**) against *L. braziliensis* and therefore in the search for an effective treatment for the stigmatizing and even potentially fatal diffuse leishmaniasis emerging out of Brazil and South America, reaching Europe and the Middle East [31]. Similarly, products TFM (**6**) and PyCrab (**17**) are interesting as they are active against *L. donovani*, which causes the visceral and fatal form of leishmaniasis [32].

Based on our collective findings, we propose a comprehensive mechanistic model that unifies our key observations into a single, synergistic cascade of events leading to parasite death. The inhibition of Fe-SOD serves as the initiating event, leading to an immediate accumulation of superoxide anion radicals (O_2_^−^) and inducing a state of heightened oxidative stress. This initial oxidative burst directly impacts central energy metabolism, as key glycolytic enzymes are highly susceptible to oxidative damage due to redox-sensitive components, such as cysteine residues or iron–sulfur clusters, within their active sites [33]. This provides a direct link between the antioxidant failure and the observed disruption of glucose metabolism.

Concurrently, this oxidative stress directly targets the mitochondrion, an organelle both central to bioenergetics and highly vulnerable to ROS [34]. The direct oxidative assault on the electron transport chain is compounded by the disruption of glycolysis, which starves the mitochondrion of its primary substrate, pyruvate. This dual pressure compromises the organelle’s ability to maintain its membrane potential, leading to the depolarization observed experimentally [35]. Crucially, a depolarized and dysfunctional mitochondrion transforms into a major endogenous source of further ROS, establishing a self-amplifying and irreversible feedback loop of damage.

In this unified model, our lead compounds orchestrate a multi-pronged assault on three interconnected pillars of parasite survival: (i) the primary antioxidant defenses (Fe-SOD), (ii) central energy production (glycolysis), and (iii) organellar integrity and function (mitochondria). This synergistic attack creates a catastrophic and inescapable cascade that culminates in parasite death, providing a robust explanation for the potent and selective leishmanicidal activity observed in this study.

## 4. Conclusions

Current treatments for leishmaniasis present several limitations, including poor patient compliance due to long treatment periods, storage limitations, the need for administration in clinical centers, and significant toxic side effects. The compounds investigated in this study offer a potential alternative, as they are easy to synthesize and store. Furthermore, these compounds exhibit adequate water solubility and acid stability, properties that may enable oral administration, representing a substantial improvement over existing therapies.

In addition, given the well-established biological roles of polyamines in cell growth, differentiation, and parasite survival, it is important to consider whether the activity of the studied compounds could be related to interference with endogenous polyamine metabolism and/or function. Although this possibility was not directly addressed in the present work, which focuses on the potential inhibition of the parasite FeSOD enzyme, it could nonetheless provide a complementary mechanistic basis for their antiparasitic effects and warrants further investigation.

The promising results obtained for compounds Pyt2QCl, Opy22, PyCrab, and TFM, together with their favorable physicochemical properties, highlight new opportunities in chemotherapy for leishmaniasis. Future studies should also include gene expression analyses of key genes involved in polyamine transport and biosynthetic pathways, which could help elucidate the molecular mechanisms underlying their activity and further support their development as antileishmanial agents.

## Figures and Tables

**Table 1:** Parasite IC_50_ concentrations for intracellular and extracellular forms, macrophage toxicity and Selectivity Indexes for *L. infantum*.

Compound Code	*L. infantum* IC_50_ (µM)		Macrophage Toxicity IC_50_ (µM)	Selectivity Index (SI)	
	Promastigote Forms	Amastigote Forms		Promastigote Forms	Amastigote Forms
Glucantime	50.1 ± 2.3	84.6 ± 4.3	175.8 ± 10.6	3.5	2
VTAL (**1**)	536.1 ± 17.8	nd	633.5 ± 21.4	1 (0)	nd
TAM (**2**)	1409.3 ± 62.7	nd	20.5 ± 1.9	0 (0)	nd
TNM (**3**)	69.2 ± 4.3	nd	348.7 ± 11.5	5 (1)	nd
TPiM (**4**)	25.57 ± 2.1	nd	33.4 ± 2.5	1 (0)	nd
TDM (**5**)	1280.3 ± 77.8	nd	1936.6 ± 83.9	2 (1)	nd
T4QT (**11**)	591.7 ± 23.7	nd	133.6 ± 6.4	0 (0)	nd
T2QT (**12**)	43.8 ± 2.1	nd	124.9 ± 7.1	3 (1)	nd
TBPZT (**9**)	32.2 ± 0.9	nd	1541.7 ± 68.7	48 (14)	nd
TFM (**6**)	32.7 ± 2.0	nd	419.8 ± 14.8	13 (3)	nd
TFLM (**7**)	350.2 ± 2.9	nd	63.0 ± 2.5	0 (0)	nd
TFT (**10**)	86.4 ± 6.8	nd	36.5 ± 3.0	0 (0)	nd
TN5I (**8**)	62.2 ± 2.6	nd	22.2 ± 0.9	0 (0)	nd
Opy22 (**13**)	134.2 ± 5.7	nd	231.5 ± 11.5	2 (1)	nd
Pyt2QCl (**15**)	68.1 ± 3.7	nd	152.1 ± 7.3	2 (1)	nd
Pyt2Ql (**16**)	15.2 ± 1.1	nd	60.9 ± 3.7	4 (1)	nd
PyCrab (**17**)	32.7 ± 1.7	nd	242.3 ± 54.6	7 (2)	nd
PZ2N2 (**18**)	6.4 ± 0.4	nd	37.5 ± 1.6	6 (2)	nd
PytF (**14**)	15.9 ± 0.8	nd	147.3 ± 8.3	9 (2)	nd
Py2M2 (**19**)	69.1 ± 2.5	nd	1914.1 ± 112.5	28 (7)	nd

IC_50_ values and calculated Selectivity Indexes for compounds tested on *L. infantum* forms. IC_50_ is the concentration required to obtain a 50% inhibition in parasite or cell population, calculated with a linear regression analysis from the employed concentrations (12.5, 25.0, 50.0 and 100.0 µM for promastigote and amastigote forms of *Leishmania infantum* and 20, 100, 500 and 5000 µM for host cells), nd meaning not determined. Selectivity Index = IC_50_ against J774.2 macrophages/IC_50_ parasite (promastigote forms). The number of times that ligand SIs exceed the reference drug SI is shown in brackets. Results represent the average of three separated determinations.

**Table 2:** Parasite IC_50_ concentrations for intracellular and extracellular forms, macrophage toxicity and Selectivity Indexes for *L. braziliensis*.

Compound Code	*L. braziliensis* IC_50_ (µM)		Macrophage Toxicity IC_50_ (µM)	Selectivity Index (SI)	
	Promastigote Forms	Amastigote Forms		Promastigote Forms	Amastigote Forms
Glucantime	51.4 ± 2.5	103.3 ± 2	175.8 ± 10.6	3	1.7
VTAL (**1**)	662.4 ± 51.6	nd	633.5 ± 21.4	1 (0)	nd
TAM (**2**)	389.4 ± 16.8	nd	20.5 ± 1.9	0 (0)	nd
TNM (**3**)	99.9 ± 3.8	nd	348.7 ± 11.5	3 (1)	nd
TPiM (**4**)	30.1 ± 1.8	nd	33.4 ± 2.5	1 (0)	nd
TDM (**5**)	1942.7 ± 99.7	nd	1936.6 ± 83.9	1 (0)	nd
T4QT (**11**)	452.1 ± 20.4	nd	133.6 ± 6.4	0 (0)	nd
T2QT (**12**)	41.3 ± 3.6	nd	124.9 ± 7.1	3 (1)	nd
TBPZT (**9**)	31.5 ± 2.0	nd	1541.7 ± 68.7	49 (16)	nd
TFM (**6**)	35.6 ± 2.1	nd	419.8 ± 14.8	12 (4)	nd
TFLM (**7**)	394.7 ± 8.9	nd	63.0 ± 2.5	0 (0)	nd
TFT (**10**)	715.1 ± 21.3	nd	36.5 ± 3.0	0 (0)	nd
TN5I (**8**)	130.1 ± 6.3	nd	22.2 ± 0.9	0 (0)	nd
Opy22 (**13**)	0.7 ± 0.1	6.3 ± 0.6	231.5 ± 11.5	331 (110)	37 (19)
Pyt2QCl (**15**)	4.3 ± 0.4	0.5 ± 0.1	152.1 ± 7.3	35 (12)	304 (152)
Pyt2Ql (**16**)	73.1 ± 2.5	nd	60.9 ± 3.7	1 (0)	nd
PyCrab (**17**)	14.9 ± 0.5	nd	242.3 ± 54.6	16 (5)	nd
PZ2N2 (**18**)	54.9 ± 2.8	nd	37.5 ± 1.6	1 (0)	nd
PytF (**14**)	8.9 ± 0.6	nd	147.3 ± 8.3	17 (6)	nd
Py2M2 (**19**)	92.4 ± 3.5	nd	1914.1 ± 112.5	21 (7)	nd

IC_50_ values and calculated Selectivity Indexes for compounds tested on *L. braziliensis* forms. IC_50_ is the concentration required to obtain a 50% inhibition in parasite or cell population, calculated with a linear regression analysis from the employed concentrations (12.5, 25.0, 50.0 and 100.0 µM for promastigote and amastigote forms of *Leishmania braziliensis* and 20, 100, 500 and 5000 µM for host cells), nd meaning not determined. Selectivity index = IC_50_ against J774.2 macrophages/IC_50_ parasite (promastigote forms). The number of times that ligand SIs exceed the reference drug SI is shown in brackets. Results represent the average of three separated determinations.

**Table 3:** Parasite IC_50_ concentrations for intracellular and extracellular forms, macrophage toxicity and Selectivity Indexes for *L. donovani*.

Compound Code	*L. donovani* IC_50_ (µM)		Macrophage Toxicity IC_50_ (µM)	Selectivity Index (SI)	
	Promastigote Forms	Amastigote Forms		Promastigote Forms	Amastigote Forms
Glucantime	23.7 ± 0.8	76.4 ± 4.1	175.8 ± 10.6	7	2
VTAL (**1**)	597.4 ± 8.4	nd	633.5 ± 21.4	1 (0)	nd
TAM (**2**)	691.3 ± 9.9	nd	20.5 ± 1.9	0 (0)	nd
TNM (**3**)	123.9 ± 6.1	nd	348.7 ± 11.5	3 (0)	nd
TPiM (**4**)	31.4 ± 1.5	nd	33.4 ± 2.5	1 (0)	nd
TDM (**5**)	1050.3 ± 77.5	nd	1936.6 ± 83.9	2 (0)	nd
T4QT (**11**)	465.6 ± 8.4	nd	133.6 ± 6.4	0 (0)	nd
T2QT (**12**)	111.2 ± 4.7	nd	124.9 ± 7.1	1 (0)	nd
TBPZT (**9**)	70.8 ± 5.3	nd	1541.7 ± 68.7	22 (3)	nd
TFM (**6**)	0.4 ± 0.1	1.5 ± 0.3	419.8 ± 14.8	1050 (150)	280 (140)
TFLM (**7**)	284.6 ± 6.3	nd	63.0 ± 2.5	0 (0)	nd
TFT (**10**)	264.9 ± 9.4	nd	36.5 ± 3.0	0 (0)	nd
TN5I (**8**)	1908.5 ± 94.3	nd	22.2 ± 0.9	0 (0)	nd
Opy22 (**13**)	311.0 ± 7.4	nd	231.5 ± 11.5	1 (0)	nd
Pyt2QCl (**15**)	14.7 ± 0.5	nd	152.1 ± 7.3	10 (1)	nd
Pyt2Ql (**16**)	14.51 ± 0.3	nd	60.9 ± 3.7	4 (1)	nd
PyCrab (**17**)	0.4 ± 0.1	5.7 ± 0.2	242.3 ± 54.6	606 (87)	43 (22)
PZ2N2 (**18**)	16.5 ± 0.7	nd	37.5 ± 1.6	2 (0)	nd
PytF (**14**)	10.2 ± 0.4	nd	147.3 ± 8.3	14 (2)	nd
Py2M2 (**19**)	208.4 ± 6.4	nd	1914.1 ± 112.5	9 (1)	nd

IC_50_ values and calculated Selectivity Indexes for compounds tested on *L. donovani* forms. IC_50_ is the concentration required to obtain a 50% inhibition in parasite or cell population, calculated with a linear regression analysis from the employed concentrations (12.5, 25.0, 50.0 and 100.0 µM for promastigote and amastigote forms of *Leishmania donovani* and 20, 100, 500 and 5000 µM for host cells), nd meaning not determined. Selectivity index = IC_50_ against J774.2 macrophages/IC_50_ parasite (promastigote forms). The number of times that ligand SIs exceed the reference drug SI is shown in brackets. Results represent the average of three separated determinations.

## Data Availability

The original contributions presented in this study are included in the article. Further inquiries can be directed to the corresponding author.

## Abbreviations

The following abbreviations are used in this manuscript:

CL	Cutaneous Leishmaniasis
ML	Mucocutaneous Leishmaniasis
VL	Visceral Leishmaniasis
tren	tris(2-aminoethyl)amine
ECACC	European Collection of Cell Cultures
MTL	Medium for Trypanosomes (Liquid)
SI	Selectivity Index
Cu/Zn-SOD	Copper/Zinc Superoxide Dismutase
Fe-SOD	Iron Superoxide Dismutase
ROS	Reactive Oxygen Species
NMR	Nuclear Magnetic Resonance
MM-MD	Molecular Mechanics-Molecular Dynamics
RMSd	Root Mean Square deviation
